# Supplementary material for: HIVprotI: an integrated web based platform for prediction and design of HIV proteins inhibitors
Source: J Cheminform. 2018 Mar 9;10:12. doi: 10.1186/s13321-018-0266-y (PMC5845081; doi:10.1186/s13321-018-0266-y)
Supplement: Supplementary file 2 — Additional file 2. Source code of HIVProtI web server. [file 13321_2018_266_MOESM2_ESM.zip › HIVprotI_Source-code/hivprotI/weka-3-6-6/doc/weka/gui/GUIChooser.html]

GUIChooser


---


|  |  |  |
| --- | --- | --- |
| **Overview** \| **Package** \| **Class** \| **Tree** \| **Deprecated** \| **Index** \| **Help** \| **Weka's home** | |  |
| **PREV CLASS**   **NEXT CLASS** | **FRAMES**    **NO FRAMES**     **All Classes** |  |
| SUMMARY: NESTED \| FIELD \| CONSTR \| METHOD | DETAIL: FIELD \| CONSTR \| METHOD |  |


---


## weka.gui Class GUIChooser

```
java.lang.Object
  java.awt.Component
      java.awt.Container
          java.awt.Window
              java.awt.Frame
                  javax.swing.JFrame
                      weka.gui.GUIChooser
```

**All Implemented Interfaces:**: java.awt.image.ImageObserver, java.awt.MenuContainer, java.io.Serializable, javax.accessibility.Accessible, javax.swing.RootPaneContainer, javax.swing.WindowConstants

---

``` public class GUIChooser extends javax.swing.JFrame ```

The main class for the Weka GUIChooser. Lets the user choose
which GUI they want to run.

**Version:**
:   $Revision: 7212 $

**Author:**
:   Len Trigg (trigg@cs.waikato.ac.nz), Mark Hall (mhall@cs.waikato.ac.nz), FracPete (fracpete at waikato dot ac dot nz)

**See Also:**: Serialized Form

---

| **Nested Class Summary** | |
| --- | --- |
| `static class` | `GUIChooser.ChildFrameSDI`             Specialized JFrame class. |

**Nested classes/interfaces inherited from class java.awt.Component**

`java.awt.Component.BaselineResizeBehavior`


**Field Summary**

**Fields inherited from class javax.swing.JFrame**

`EXIT_ON_CLOSE`

**Fields inherited from class java.awt.Frame**

`CROSSHAIR_CURSOR, DEFAULT_CURSOR, E_RESIZE_CURSOR, HAND_CURSOR, ICONIFIED, MAXIMIZED_BOTH, MAXIMIZED_HORIZ, MAXIMIZED_VERT, MOVE_CURSOR, N_RESIZE_CURSOR, NE_RESIZE_CURSOR, NORMAL, NW_RESIZE_CURSOR, S_RESIZE_CURSOR, SE_RESIZE_CURSOR, SW_RESIZE_CURSOR, TEXT_CURSOR, W_RESIZE_CURSOR, WAIT_CURSOR`

**Fields inherited from class java.awt.Component**

`BOTTOM_ALIGNMENT, CENTER_ALIGNMENT, LEFT_ALIGNMENT, RIGHT_ALIGNMENT, TOP_ALIGNMENT`

**Fields inherited from interface javax.swing.WindowConstants**

`DISPOSE_ON_CLOSE, DO_NOTHING_ON_CLOSE, HIDE_ON_CLOSE`

**Fields inherited from interface java.awt.image.ImageObserver**

`ABORT, ALLBITS, ERROR, FRAMEBITS, HEIGHT, PROPERTIES, SOMEBITS, WIDTH`


**Constructor Summary**

`GUIChooser()`
  
          Creates the experiment environment gui with no initial experiment


| **Method Summary** | |
| --- | --- |
| `void` | `addChildFrame(java.awt.Container c)`             adds the given child frame to the list of frames. |
| `static void` | `createSingleton()`             Create a singleton instance of the GUIChooser |
| `static GUIChooser` | `getSingleton()`             Get the singleton instance of the GUIChooser |
| `static void` | `main(java.lang.String[] args)`             Tests out the GUIChooser environment. |
| `boolean` | `removeChildFrame(java.awt.Container c)`             tries to remove the child frame, it returns true if it could do such. |
| `void` | `showExplorer(java.lang.String fileToLoad)` |
| `void` | `showKnowledgeFlow(java.lang.String fileToLoad)` |

**Methods inherited from class javax.swing.JFrame**

`getAccessibleContext, getContentPane, getDefaultCloseOperation, getGlassPane, getGraphics, getJMenuBar, getLayeredPane, getRootPane, getTransferHandler, isDefaultLookAndFeelDecorated, remove, repaint, setContentPane, setDefaultCloseOperation, setDefaultLookAndFeelDecorated, setGlassPane, setIconImage, setJMenuBar, setLayeredPane, setLayout, setTransferHandler, update`

**Methods inherited from class java.awt.Frame**

`addNotify, getCursorType, getExtendedState, getFrames, getIconImage, getMaximizedBounds, getMenuBar, getState, getTitle, isResizable, isUndecorated, remove, removeNotify, setCursor, setExtendedState, setMaximizedBounds, setMenuBar, setResizable, setState, setTitle, setUndecorated`

**Methods inherited from class java.awt.Window**

`addPropertyChangeListener, addPropertyChangeListener, addWindowFocusListener, addWindowListener, addWindowStateListener, applyResourceBundle, applyResourceBundle, createBufferStrategy, createBufferStrategy, dispose, getBufferStrategy, getFocusableWindowState, getFocusCycleRootAncestor, getFocusOwner, getFocusTraversalKeys, getGraphicsConfiguration, getIconImages, getInputContext, getListeners, getLocale, getModalExclusionType, getMostRecentFocusOwner, getOwnedWindows, getOwner, getOwnerlessWindows, getToolkit, getWarningString, getWindowFocusListeners, getWindowListeners, getWindows, getWindowStateListeners, hide, isActive, isAlwaysOnTop, isAlwaysOnTopSupported, isFocusableWindow, isFocusCycleRoot, isFocused, isLocationByPlatform, isShowing, pack, paint, postEvent, removeWindowFocusListener, removeWindowListener, removeWindowStateListener, reshape, setAlwaysOnTop, setBounds, setBounds, setCursor, setFocusableWindowState, setFocusCycleRoot, setIconImages, setLocationByPlatform, setLocationRelativeTo, setMinimumSize, setModalExclusionType, setSize, setSize, setVisible, show, toBack, toFront`

**Methods inherited from class java.awt.Container**

`add, add, add, add, add, addContainerListener, applyComponentOrientation, areFocusTraversalKeysSet, countComponents, deliverEvent, doLayout, findComponentAt, findComponentAt, getAlignmentX, getAlignmentY, getComponent, getComponentAt, getComponentAt, getComponentCount, getComponents, getComponentZOrder, getContainerListeners, getFocusTraversalPolicy, getInsets, getLayout, getMaximumSize, getMinimumSize, getMousePosition, getPreferredSize, insets, invalidate, isAncestorOf, isFocusCycleRoot, isFocusTraversalPolicyProvider, isFocusTraversalPolicySet, layout, list, list, locate, minimumSize, paintComponents, preferredSize, print, printComponents, remove, removeAll, removeContainerListener, setComponentZOrder, setFocusTraversalKeys, setFocusTraversalPolicy, setFocusTraversalPolicyProvider, setFont, transferFocusBackward, transferFocusDownCycle, validate`

**Methods inherited from class java.awt.Component**

`action, add, addComponentListener, addFocusListener, addHierarchyBoundsListener, addHierarchyListener, addInputMethodListener, addKeyListener, addMouseListener, addMouseMotionListener, addMouseWheelListener, bounds, checkImage, checkImage, contains, contains, createImage, createImage, createVolatileImage, createVolatileImage, disable, dispatchEvent, enable, enable, enableInputMethods, firePropertyChange, firePropertyChange, firePropertyChange, firePropertyChange, firePropertyChange, firePropertyChange, getBackground, getBaseline, getBaselineResizeBehavior, getBounds, getBounds, getColorModel, getComponentListeners, getComponentOrientation, getCursor, getDropTarget, getFocusListeners, getFocusTraversalKeysEnabled, getFont, getFontMetrics, getForeground, getHeight, getHierarchyBoundsListeners, getHierarchyListeners, getIgnoreRepaint, getInputMethodListeners, getInputMethodRequests, getKeyListeners, getLocation, getLocation, getLocationOnScreen, getMouseListeners, getMouseMotionListeners, getMousePosition, getMouseWheelListeners, getName, getParent, getPeer, getPropertyChangeListeners, getPropertyChangeListeners, getSize, getSize, getTreeLock, getWidth, getX, getY, gotFocus, handleEvent, hasFocus, imageUpdate, inside, isBackgroundSet, isCursorSet, isDisplayable, isDoubleBuffered, isEnabled, isFocusable, isFocusOwner, isFocusTraversable, isFontSet, isForegroundSet, isLightweight, isMaximumSizeSet, isMinimumSizeSet, isOpaque, isPreferredSizeSet, isValid, isVisible, keyDown, keyUp, list, list, list, location, lostFocus, mouseDown, mouseDrag, mouseEnter, mouseExit, mouseMove, mouseUp, move, nextFocus, paintAll, prepareImage, prepareImage, printAll, removeComponentListener, removeFocusListener, removeHierarchyBoundsListener, removeHierarchyListener, removeInputMethodListener, removeKeyListener, removeMouseListener, removeMouseMotionListener, removeMouseWheelListener, removePropertyChangeListener, removePropertyChangeListener, repaint, repaint, repaint, requestFocus, requestFocusInWindow, resize, resize, setBackground, setComponentOrientation, setDropTarget, setEnabled, setFocusable, setFocusTraversalKeysEnabled, setForeground, setIgnoreRepaint, setLocale, setLocation, setLocation, setMaximumSize, setName, setPreferredSize, show, size, toString, transferFocus, transferFocusUpCycle`

**Methods inherited from class java.lang.Object**

`equals, getClass, hashCode, notify, notifyAll, wait, wait, wait`

**Methods inherited from interface java.awt.MenuContainer**

`getFont, postEvent`

**Constructor Detail**

### GUIChooser

```
public GUIChooser()
```

:   Creates the experiment environment gui with no initial experiment


**Method Detail**

### createSingleton

```
public static void createSingleton()
```

:   Create a singleton instance of the GUIChooser

---


### getSingleton

```
public static GUIChooser getSingleton()
```

:   Get the singleton instance of the GUIChooser

    :   **Returns:**: the singleton instance of the GUIChooser

---


### showKnowledgeFlow

```
public void showKnowledgeFlow(java.lang.String fileToLoad)
```

---


### showExplorer

```
public void showExplorer(java.lang.String fileToLoad)
```

---


### addChildFrame

```
public void addChildFrame(java.awt.Container c)
```

:   adds the given child frame to the list of frames.

    :   **Parameters:**: `c` - the child frame to add

---


### removeChildFrame

```
public boolean removeChildFrame(java.awt.Container c)
```

:   tries to remove the child frame, it returns true if it could do such.

    :   **Parameters:**: `c` - the child frame to remove **Returns:**: true if the child frame could be removed

---


### main

```
public static void main(java.lang.String[] args)
```

:   Tests out the GUIChooser environment.

    :   **Parameters:**: `args` - ignored.


---


|  |  |  |
| --- | --- | --- |
| **Overview** \| **Package** \| **Class** \| **Tree** \| **Deprecated** \| **Index** \| **Help** \| **Weka's home** | |  |
| **PREV CLASS**   **NEXT CLASS** | **FRAMES**    **NO FRAMES**     **All Classes** |  |
| SUMMARY: NESTED \| FIELD \| CONSTR \| METHOD | DETAIL: FIELD \| CONSTR \| METHOD |  |


---
